# A Deep Sparse Capsule Network for Non-Invasive Blood Glucose Level Estimation Using a PPG Sensor

**DOI:** 10.3390/s25061868

**Published:** 2025-03-18

**Authors:** Narmatha Chellamani, Saleh Ali Albelwi, Manimurugan Shanmuganathan, Palanisamy Amirthalingam, Emad Muteb Alharbi, Hibah Qasem Salman Alatawi, Kousalya Prabahar, Jawhara Bader Aljabri, Anand Paul

**Affiliations:** 1 Faculty of Computers and Information Technology, University of Tabuk, Tabuk 71491, Saudi Arabia; sbalawi@ut.edu.sa (S.A.A.); mmurugan@ut.edu.sa (M.S.); emalharbi@ut.edu.sa (E.M.A.); hq_alatawi@ut.edu.sa (H.Q.S.A.); jalamri@ut.edu.sa (J.B.A.); 2 Department of Pharmacy Practice, Faculty of Pharmacy, University of Tabuk, Tabuk 71491, Saudi Arabia; pchettiar@ut.edu.sa (P.A.); kgopal@ut.edu.sa (K.P.); 3 Biostatistics & Data Science, LSU Health Sciences Center New Orleans, New Orleans, LA 70112, USA; apaul4@lsuhsc.edu

**Keywords:** PPG sensor, blood glucose level, DSCNet, deep learning, non-invasive

## Abstract

Diabetes, a chronic medical condition, affects millions of people worldwide and requires consistent monitoring of blood glucose levels (BGLs). Traditional invasive methods for BGL monitoring can be challenging and painful for patients. This study introduces a non-invasive, deep learning (DL)-based approach to estimate BGL using photoplethysmography (PPG) signals. Specifically, a Deep Sparse Capsule Network (DSCNet) model is proposed to provide accurate and robust BGL monitoring. The proposed model’s workflow includes data collection, preprocessing, feature extraction, and predictions. A hardware module was designed using a PPG sensor and Raspberry Pi to collect patient data. In preprocessing, a Savitzky–Golay filter and moving average filter were applied to remove noise and preserve pulse form and high-frequency components. The DSCNet model was then applied to predict the sugar level. Two models were developed for prediction: a baseline model, DSCNet, and an enhanced model, DSCNet with self-attention. DSCNet’s performance was evaluated using Mean Absolute Percentage Error (MAPE), Mean Absolute Error (MAE), Mean Squared Error (MSE), Root Mean Squared Error (RMSE), Mean Absolute Relative Difference (MARD), and coefficient of determination (R^2^), yielding values of 3.022, 0.05, 0.058, 0.062, 10.81, and 0.98, respectively.

## 1. Introduction

Glucose is a primary and efficient energy source for the human body. Maintaining a normal blood glucose level (BGL) between 80 and 150 mg/dL is essential for carrying out daily activities. However, deviations from this range can lead to various physiological issues. Insulin, a vital hormone produced by the pancreas, helps regulate glucose levels in the bloodstream following food intake. Insufficient insulin production causes glucose to accumulate to toxic levels in the blood. When the proliferation of alpha cells exceeds that of beta cells, it can lead to a sustained increase in the BGL, impairing the liver’s ability to produce enough insulin to balance glucose levels. Diabetes mellitus is a chronic metabolic condition resulting from a persistently high BGL (above the normal range) [1]. It is characterized by a BGL exceeding 230 mg/dL or dropping below 65 mg/dL. Individuals with diabetes have an impaired ability to produce or use insulin effectively, which is essential for facilitating glucose absorption by cells for energy. The global prevalence of diabetes is substantial and steadily increasing. The World Health Organization (WHO) estimates that the global diabetic population (ages 18–99) will rise from 451 million in 2017 to 693 million by 2045. This surge is anticipated to cause a 53% increase in annual diabetes-related expenditures, encompassing both medical and non-medical costs, projected to grow from USD 407.6 billion in 2015 to over USD 622.3 billion by 2030. Therefore, accurate and consistent BGL monitoring is crucial for effective diabetes management [2].

Systematic monitoring of BGLs can help reduce the occurrence of complications associated with diabetes. There are three main categories of blood component level measurement techniques: invasive, non-invasive, and minimally invasive. Invasive blood glucose detection technology, which involves collecting blood samples and then analyzing them in vitro for blood glucose measurement, is used by both hospitals and home glucometers due to its widespread use, convenience, and practicality. While this approach yields accurate results and serves as a crucial foundation for diabetes diagnosis, it is not suitable for ongoing monitoring of diabetes because of its tedious procedure, extended detection time, and substantial volume of vein blood extracted. Non-invasive BGL monitoring is the measurement of human BGLs without causing harm to physiological tissues. Non-invasive blood glucose detection encompasses several techniques, which can be broadly categorized as optical, microwave, and electrochemical technologies [3].

An estimation of blood component levels using the non-invasive model simply requires a bio-signal, such as an image or spectrum. Non-invasive technology has emerged as a more widely used approach in smart healthcare research, effectively addressing the issue. Glucose detection technologies can be categorized into four sub-groups: optical, electrical, thermal, and nanotechnology methodologies. An optical technique refers to all the methods that have been created for operation in the infrared and the optical regions of the spectrum. These approaches use the absorption, reflection, and scattering characteristics of light as it traverses biological media [4]. PPG is the predominant method for non-invasively measuring blood component levels in present times. The significance of the PPG signal lies in its accessibility through non-invasive and cost-effective sensors. It is a method widely used in medical settings to quantify the saturation level of oxygen (SpO_2_) in the bloodstream and the pulse rate, which serves as a crucial indicator of the patient’s health. The equipment employed for capturing PPG signals is characterized by its simplicity and affordability. This device comprises two essential components: a light-emitting diode (LED) and a photodiode. The LED functions as a laser or light source (emitter), while the photodiode acts as a light detector (receiver). Photoelectric methods can be used to record PPG signals from the fingers, nasal septum, and toes [5].

**Figure 1:**
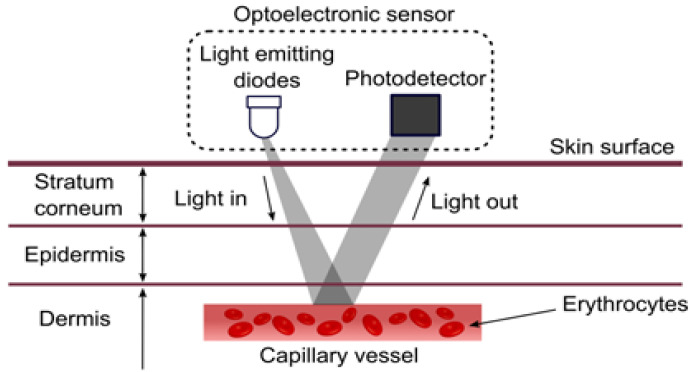
Working principle of PPG sensors [6].

In transmission mode, a PPG sensor allows LED light to traverse absorbent materials, including bone, arterial and venous blood, and skin pigmentation. The detector then receives the light and quantifies it using filters and converters. Conversely, a PPG sensor operating in a reflection course reflects the fluorescent light emitted by an LED onto the skin. This light is then tracked by the detector and measured using filters and converters in a similar manner [6]. The operational mechanism of the sensor relies on the emission of infrared radiation with an LED that can efficiently pass through the skin and blood vessels [7]. The detector captures this light to quantify the blood flow, as visualized in Figure 1.

This work aims to achieve the non-invasive estimation of BGLs using a PPG sensor. The primary objective is to construct a BGL monitoring system utilizing the PPG sensor by integrating machine learning (ML) or deep learning (DL) techniques. ML refers to the integration of many computer techniques that can be improved through experimental testing and data exploitation. The algorithms construct a model that depends on a set of sample data, known as training data, to facilitate prediction or decision-making [8]. This work introduces a new non-invasive technique based on DL to compute the BGL using PPG signals. The rising prevalence of diabetes and related health complications has underscored the need for effective and non-invasive methods of blood glucose monitoring. Traditional methods, which often rely on invasive techniques, can be painful and inconvenient and lead to poor patient compliance. Current technologies, including Continuous Glucose Monitoring (CGM) devices, while effective, often come with high costs and require extensive calibration. Consequently, there is a pressing need for innovative solutions that offer accurate, non-invasive, and user-friendly blood glucose monitoring systems. This research aims to address the urgent need for a reliable and non-invasive blood glucose monitoring solution that utilizes PPG signals combined with advanced algorithms. The scope includes the design and implementation of a user-friendly model capable of capturing PPG signals, the evaluation and predictions using the DL algorithm for effective data analysis, and rigorous testing across diverse patient demographics to ensure the system’s accuracy and reliability [9,10].

Traditional models encounter difficulties in maintaining constant accuracy in predicting glucose levels, especially in patients with varying health problems or in dynamic environments. Many existing models exhibit weak preprocessing procedures, generally failing to properly eliminate noise while maintaining essential signal features. This leads to poor signal quality, hence reducing the accuracy of glucose level measurement. PPG signals provide a continuous, cost-effective, and non-invasive monitoring solution that enables real-time glucose level tracking without the risks associated with traditional techniques. Furthermore, few studies have included advanced deep learning methodologies such as attention mechanisms, which could greatly enhance the model’s capacity for focusing on essential PPG signal characteristics while reducing the influence of unrelated information. This research introduces a novel Deep Sparse Capsule Network (DSCNet) that incorporates sophisticated noise reduction techniques, including the Savitzky–Golay and moving average filters, to improve the quality of PPG data. The integration of self-attention mechanisms allows the model to emphasize essential aspects, enhancing the robustness and accuracy of glucose level predictions.

This research advances blood glucose monitoring by introducing a novel DSCNet model. The model utilizes sensor data obtained from a PPG sensor to reliably monitor glucose levels. It provides real-time monitoring and specific measures to enhance patient outcomes, relying on the PPG sensor. The proposed approach offers a more robust and efficient solution for blood glucose level monitoring in various healthcare environments by using advanced feature extraction and DL-based classification methods. The main contributions of this research are summarized as follows:

- Developed a novel DSCNet model for monitoring blood glucose levels (BGL) using photoplethysmography (PPG) signals and utilized a PPG sensor device for real-time sensing and monitoring of BGLs.
- Enhanced preprocessing techniques by integrating the Savitzky–Golay filter and moving average filter, resulting in improved signal quality.
- Improved the accuracy of the model through advanced feature extraction techniques, including Autoregressive (AR), Kaiser Teager energy (KTE), and spectral entropy (SE), among others.
- Evaluated the DSCNet model’s performance using actual PPG sensor data and assessed its effectiveness through various metrics, including Mean Absolute Percentage Error (MAPE) and Mean Squared Error (MSE), to compare and evaluate the performance of the DSCNet model with existing approaches.

The subsequent sections of this research are structured as follows: Section 2 presents an analysis of the existing non-invasive BGL monitoring models. Section 3 provides the implementation of the proposed hardware and presents the DSCNet model. Section 4 includes the discussion of experimental analysis and the comparison of findings. Section 5 concludes the work and suggests the possible areas for further research.

## 2. Related Works

Susana et al. presented a machine learning model to classify BGL non-invasively for the early detection of diabetes using a PPG signal. The PPG signal was obtained by a non-invasive finger sensor. The classification of each PPG signal was determined by matching the findings of the glucometer test with the label “normal” or “diabetes”. A single PPG data segment comprised 2100 sample points. Upon the completion of data collection, the datasets were partitioned to validate the classifier’s accuracy. The ensemble bagged trees algorithm (EBTA) was devised to enhance the diagnostic accuracy of classification algorithms. Achieving superior accuracy, the EBTA outperformed several ML algorithms, namely K-nearest neighbor (KNN), RUS boosted trees, subspace KNN, decision trees, Gaussian SVM, EBT, support vector machine (SVM), bagged trees, support vector regression quadratic, and fine Gaussian support vector regression (FGSVR). In the testing of the models, the EBT approach demonstrated superior performance [11]. Gupta et al. developed an innovative non-invasive PPG system to monitor essential health indicator parameters. The fingertip PPG device had both transmitting and reflecting data gathering systems, which were activated by illuminating the skin with green, red, and infrared (IR) LEDs. The preprocessing method employed was the fitting-based sliding window (FSW) approach. The feature extraction model retrieved many domain features and utilized two ML models (XGBoost and random forest (RF)) to align the features of the training sample with the real prediction of BGLs. Between the two regression models, XGB outperforms RF. The estimation of the BGL with an acceptable degree of clinical precision was performed by XGB [12].

An approach for the measurement of human blood components from fingertip video utilizing Deep Neural Networks (DNNs) for PPG signals was presented by Haque et al. [13]. Data were gathered from 93 participants via fingertip videos captured on a smartphone. The PPG signal, along with its first and second derivatives, and Fourier analysis were used to extract 46 distinctive features. A correlation-based feature selection approach utilizing a genetic algorithm to choose suitable characteristics was employed. Novel DNN models were developed to predict the concentrations of blood hemoglobin (Hb), creatinine (Cr), and glucose (Gl) using certain features. The technique yielded the highest estimation accuracy of R^2^. A novel smartphone video-based non-invasive method was developed by Islam et al. [14] for the quantitative estimation of blood glucose levels. Video data were systematically gathered from a patient’s fingertip using cameras in a smartphone and then transformed into a PPG signal. To eliminate optical noise, motion interference, and high-frequency noise from the PPG data, a Gaussian filter was implemented using the Asymmetric Least Square (ALS) approach.

These preprocessed signals were further utilized to extract signal characteristics like diastolic and systolic peaks, the temporal intervals between successive peaks, and the first derivative and second derivative peaks. To predict glucose levels, the characteristics were inputted into regression models. The PLS model exhibited the lowest standard error of prediction (SEP) among the statistical learning methods for an unbiased dataset. The implementation of empirical mode decomposition (EMD- based non-invasive BGL measurement from PPG signals using ML algorithms was demonstrated by Satter et al. [15]. A PPG signal was obtained using a wrist device and subjected to preprocessing procedures. The EMD method was used to extract features from PPG signals to estimate BGL. As input to the regression algorithm, both IMF-based and PPG-waveform-based features were used. After the selection of features, 50 features were used to predict the BGL. CatBoost consistently showed superior performance compared to LightGBM, XGB, and RF over a diverse range of attributes.

A CNN with attention mechanism and bidirectional LSTM (ACNN-BiLSTM) model for non-invasive BP assessment utilizing multi-wavelength (MV) PPG was developed by Cui et al. [16]. The integration of continuous wavelet transform (CWT) within an MV PPG fusion technique was employed to address the constraints in blood pressure (BP) prediction. Following the applications of signal filtering and outlier removal, the preprocessed four-channel data underwent separate CWT transformations and were then included in the dataset as images. The 4-channel CWT images were combined into the 12-channel array and utilized as input for the DL model for classification. The BP prediction performance was assessed using the 10-fold cross-validation approach. The results demonstrated that the model outperformed the multi-wavelength PPG fusion approach in terms of MAE and RMSE.

An architectural design of a personalized glucose monitoring system (PGMS) was introduced by Anand et al. [17]. Initially, blood glucose levels were assessed using both invasive and non-invasive methods to train the ML algorithms. The paired data and associated errors were systematically categorized into six distinct clusters according to the blood glucose ranges reflecting the patient’s diabetic circumstances. The PGMS strategy employed the AdaBoost algorithm and domain knowledge clustering technique to effectively train error prediction models. The validity of the PGMS was confirmed by evaluation using two distinct datasets. On dataset 1 and dataset 2, the PGMS obtained a better MARD. The work by Hina et al. [18] presented a wearable device that utilizes near-infrared (NIR) PPG with a single wavelength in conjunction with ML regression (MLR) to monitor glucose levels. The PPG readout circuit comprised a transimpedance amplifier with a gain of 1 MΩ and a 10 Hz low-pass filter (LPF) switching capacitor. The digital processor achieved motion artifact and baseline drift elimination from PPG data, extracted six distinct characteristics, and ultimately predicted the BGL using FGSVR with MLR. The entire system was constructed using a CMOS process. The technique produced a precise prediction of the sugar level, achieving a better MARD.

A non-invasive method for BGL measurement with PPG using time–frequency Analysis was initially proposed by Susana et al. [19]. The data collection was generated utilizing the Guilin People’s Hospital comprehensive open database. Time–frequency (TF) analysis was used to modify the frequency domain information of PPG data by incorporating the spectral entropy (SE) and instantaneous frequency (IF). The study utilized three distinct types of raw data as inputs: the raw signal from PPG, the signal with IF, and SE. SVM achieved a higher degree of accuracy in comparison to subspace decision trees and KNN. An ML model was developed by Agrawal et al. [20] for BGL measurement utilizing a non-invasive technique on two distinct datasets. ML algorithms were implemented on two datasets: the PIDD and iGLU datasets. The Clarke error grid was examined, and all values were found to be within zones ‘A’ and ‘B’, indicating that the model accurately predicted diabetes. Comparative analysis of the algorithms was conducted using the RMSE, MAE, accuracy, precision, ROC, F-1 measure, and recall metrics derived from the dataset. With an AUC value, the RF and LR models demonstrated strong performance in detecting diabetes.

Furthermore, a decision tree was employed to predict glucose levels with a higher level of precision. The prediction of non-invasive BGL measurement using DL with cohort arrangement was described by Chu et al. [21]. A model was developed using a sample size of 2538 patients, who were divided into two groups: with and without medication. An additional characteristic included in the training data was the quarterly measured glycated hemoglobin HbA1c. A one-dimensional CNN employed both micro and macro training. The top-performing model for individuals without medication, using quarterly recorded HbA1c, attained a high prediction accuracy. A method for estimating glycated hemoglobin combining ML with PPG was proposed by Kwon et al. [22]. Two widely used ensemble techniques in ML were investigated: RF and XGB. In the preprocessing time, a sixth-order Butterworth lowpass filter was employed to eliminate high-frequency noise. Moreover, a total of 18 features were retrieved, and 7 features exhibiting the highest performances were chosen by feature importance assessment. With performance comparisons of diabetes status diagnosis and error analysis, the superiority of the ML approach was shown. Overall, XGB exhibited superior performance compared to RF. A non-invasive BGL estimation system that utilized bioelectrical impedance measuring and a neural network with dual-wavelength PPG was introduced by Yen et al. [23]. A BPNN with weight adjustment and a broad application level was employed as the BGL prediction model. To strengthen the resilience of the network model, the data preprocessing stage incorporated SG filtering to overcome baseline drift induced by motion artifacts [24,25,26,27,28].

Existing studies in non-invasive BGL monitoring using PPG signals face several limitations. Many methods rely on extensive preprocessing and are often unable to adapt effectively to diverse datasets. Advanced models such as XGBoost and regression techniques have shown promise but struggle with issues like noise interference, limited real-time applicability, and computational complexity. Techniques involving feature extraction or multi-wavelength PPG signals demonstrate high accuracy but are resource-intensive and require specialized sensor setups, making them less practical for widespread use. Additionally, models utilizing deep learning often face challenges with noise resilience and scalability. To address these gaps, this research introduces a Deep Sparse Capsule Network (DSCNet) with self-attention mechanisms, offering improved noise robustness, reduced computational demands, and enhanced scalability. Combined with efficient preprocessing techniques like Savitzky–Golay and moving average filters, the proposed method provides a comprehensive and practical solution for non-invasive BGL monitoring.

The computational complexity of bioelectrical impedance processing was reduced by dimensionality reduction utilizing Principal Component Analysis (PCA) to preserve essential information. The model’s predicted blood glucose readings were evaluated using the following indicators: MSE, RMSE, MAE, MARD, and R^2^. A model for the non-invasive estimation of hemoglobin and glucose levels using PPG fingertip video using a multigene genetic programming (MGGP)-based approach was presented by Golap et al. [29]. A total of 111 fingertip videos were gathered from patients of different types. To remove noise during the preprocessing stage, a finite impulse response filter was implemented. A correlation-based feature selection approach utilizing a genetic algorithm was utilized to choose the optimal features. The constructed model underwent training using regression techniques including LR, SVR, and RFR. In comparison to traditional regression approaches, the MGGP-based symbolic regression model exhibited superior accuracy.

The estimation of BGLs via PPG on the fingertip using a Monte Carlo photon (MCP) simulation-based model was introduced by Haque et al. [30]. The bio-optical characteristics of the finger model were derived to develop the method for a layer of the fingers. The intensities of the observed photons obtained from simulations with the model were utilized to calculate the BGL utilizing an XGB algorithm. The XGB model underwent training using synthetic data acquired from the MCP simulations and was subsequently evaluated using both synthetic and actual data. The model’s Pearson correlation coefficient was obtained high during testing with synthetic and actual data. Table 1 summarizes the existing models.

The reviewed studies demonstrate the range of innovative approaches to non-invasive BGL monitoring using PPG and various ML techniques. Techniques include the EBTA achieving better accuracy in classification, XGBoost models outperforming RF in clinical settings, and DNN providing high estimation accuracy for blood components. Notable methods involve smartphone-based PPG systems, time–frequency analysis, and dual-wavelength PPG with neural networks. However, despite these advancements, significant research gaps persist. There is a need for more robust validation across diverse populations, improved handling of data variability and noise, and integration of multimodal sensing to enhance accuracy and reliability. Additionally, while models like XGB and DNN have shown promise, their generalizability across different conditions and populations remains underexplored. Addressing these gaps could further enhance the effectiveness and applicability of non-invasive BGL monitoring technologies.

## 3. Proposed Research Methodology

**Figure 2:**
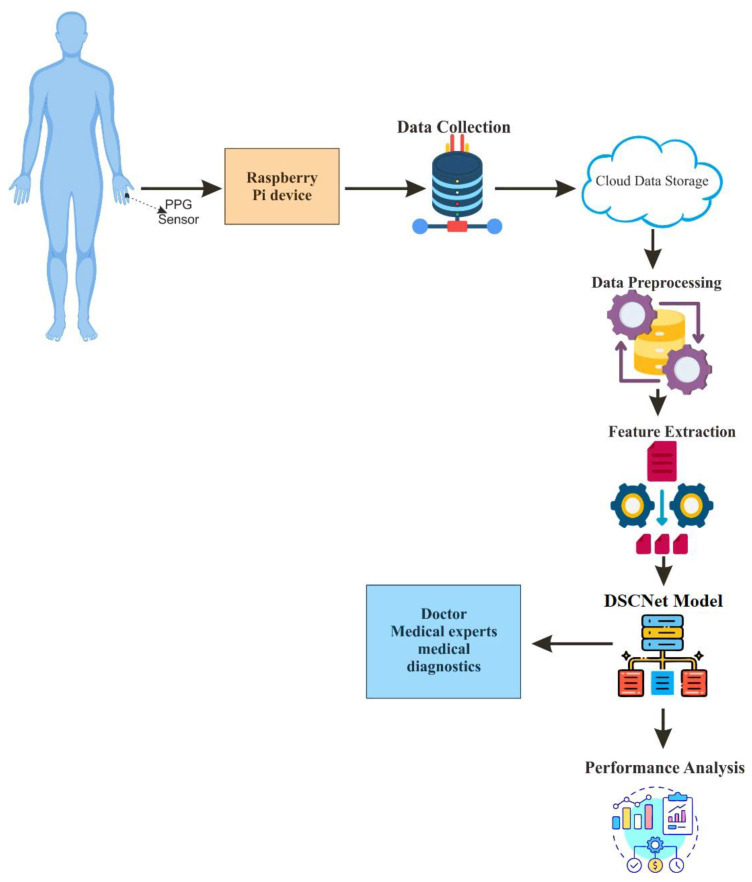
Proposed system architecture.

This research proposes a DL-model to monitor the BGL using PPG. The proposed approach is specifically developed to provide a non-invasive, precise, painless, and highly accurate solution. The research model’s architecture is depicted in Figure 2. The hardware module, along with a PPG sensor and Raspberry Pi computer, is specifically built to gather data from multiple patients. A PPG sensor is utilized to obtain PPG signals from patients. The key element in this research is the PPG signal, acquired by the PPG sensor from a patient’s finger. Once the finger is placed over the device, the sensor collects the PPG signals. The Raspberry Pi device is employed to transmit signals acquired by the PPG sensor to the cloud utilizing the ESP-32-WROOM-32UE module. The data stored in the cloud are fed as input to the proposed DL model for monitoring the BGL.

The suggested approach encompasses a sequence of tasks, including the preprocessing of data, extraction of features, and predictions. Data preprocessing is an essential initial stage before the implementation of any analysis or modeling methods on the data. The filters called the moving average filter and Savitzky–Golay filter are used in preprocessing to remove baseline wandering and preserve the pulse shape and high-frequency components of the data, respectively. After preprocessing, feature extraction is performed by using several techniques, namely AR, KTE, HRS, SES, EP, PTT, PPI, and PA. The efficacy of this proposed model will be evaluated and compared to other existing methods.

### 3.1. Hardware Specifications

**Figure 3:**
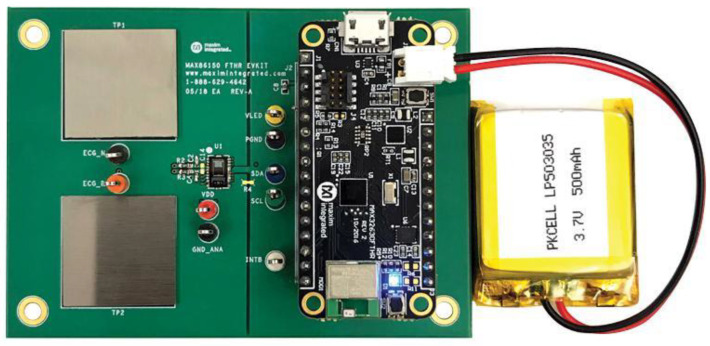
PPG sensor board [6].

PPG Sensor: A PPG signal is acquired by optically quantifying the variations in blood volume throughout the tissue of the skin, while an electrocardiogram (ECG) sensor may identify the heart’s electrical activity. An outstanding characteristic of this chip is its ability to conduct simultaneous sampling of PPG and ECG signals, leading to synchronized results for both. The board includes the Arduino library and is compatible with all processors equipped with an I2C interface. The device stores the digital output data in a 32-deep First-In, First-Out (FIFO) storage strategy. This device combines the PPG and ECG sensor, pulse oximeter (SPO_2_), and heart rate sensor modules into a single integrated circuit. A visual illustration of the PPG sensor board is depicted in Figure 3.

To facilitate access, the optical sensor is located directly on the top of the board. Furthermore, a transparent epoxy resin is used to shield the optical sensor from any electrical noise caused by physical contact. The product specifications include a supply voltage of 1.8 V for the chip and 3.3 V for the LED drivers, integrated optical modules fitted with IR and red emitters and detectors, and ambient light cancellation for enhancing the signal-to-noise ratio (SNR). With the previously mentioned characteristics, this board is most suitable for use in mobile health monitoring and wearable electronic devices.

**Figure 4:**
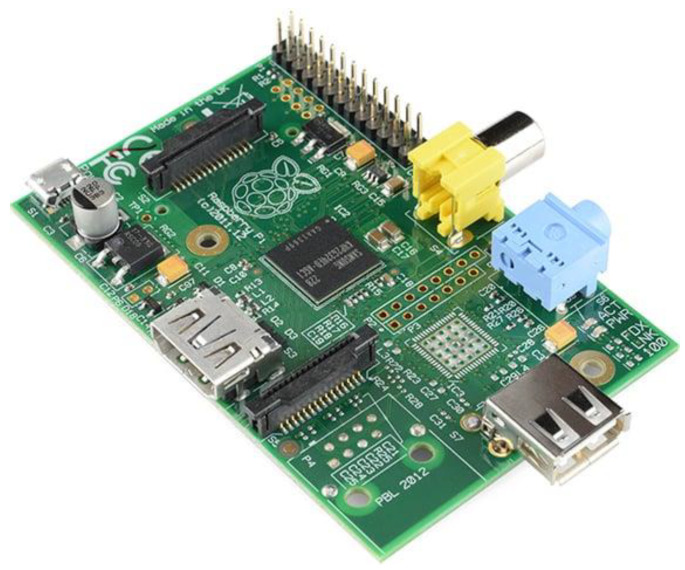
Hardware design of Raspberry Pi [31].

Raspberry Pi: The Raspberry Pi is a compact and cost-effective single-board minicomputer created by the Raspberry Pi Foundation (Cambridge, UK), a charitable organization based in the United Kingdom. The use of Raspberry Pi for glucose monitoring typically involves integrating it with sensors and software to measure and analyze blood glucose levels. The hardware design of the Raspberry Pi is shown in Figure 4. The board includes a MicroSD card configured with the Raspberry Pi OS, a micro-USB power source, and a dedicated Raspberry Pi Zero camera cable. The specifications of the board are listed as 802.11 b/g/n wireless LAN, 1 GHz, single-core CPU, 512 MB RAM, Bluetooth 4.1, composite video, HAT-compatible 40-pin header, and reset headers and CSI camera connector.

### 3.2. Data Collection

The King Fahad Specialist Hospital, Tabuk, Saudi Arabia, granted approval for the collection of samples used in this research. A total of 835 subjects participated in the collection of samples. Every participant was well briefed and explicitly agreed to the gathering and subsequent utilization of data. Two successive one-minute segments of PPG signals were captured for each participant, together with their physiological data including age, weight, BGL, and a questionnaire assessing any current medical interventions. The PPG signals were acquired by sampling at a frequency of 115.2 Hz. The invasive data were then calculated as the mean of two recordings within a predetermined interval. Every segment of the PPG data has 1700 sample points. The full duration of the experiment was around 15 min. Each PPG data collection session lasted approximately 3 min. Participants were instructed to undergo a blood glucose test in the early morning without eating and then again two hours after eating. In circumstances when patients were unaware of their diabetes status, they were also instructed to consume 75 g of glucose-infused water. The blood glucose level was categorized into three categories including values that vary from 3.9 mmol/L to 11 mmol/L. The fasting and non-fasting samples were collected in a specific interval of time. The fasting samples were collected from subjects who fasted overnight after dinner (roughly 8 to 10 h). The non-fasting samples were collected from subjects who had breakfast after a time (roughly 1 to 2 h). The corresponding levels considered for the blood glucose level in this research are also listed in Table 2. The samples of the collected data are presented in Table 3. The 1700 samples were split into an 80:20 ratio for training and testing. Algorithm 1 presents the PPG signal data collection and analysis.
**Algorithm 1.** PPG Signal Data Collection and Analysis1: Initialize parameters 2: SET frequency = 115.2 Hz 3: SET total subjects = 835 4: SET total samples per segment = 1700 5: SET segments per participant = 2 6: SET sample duration = 1 min 7: SET total duration = 15 min 8: SET fasting duration = 2 h after eating 9: SET glucose intake = 75 g of glucose-infused water 10: Data collection process 11: for each participant FROM 1 to total subjects do 12:    COLLECT participant data 13:   DISPLAY “Please agree to participate in the data collection” 14:    for segment number FROM 1 to segments per participant do 15:    RECORD PPG signal (segment number) 16:    end for 17:    participant data.age = GET age 18:    participant data.weight = GET weight 19:    participant data.blood glucose level = GET BGL(fasting) 20:    if participant is unaware of diabetes, then 21:      ADMINISTER glucose intake 22:    participant data. Blood glucose level = GET BGL(non-fasting) 23:    end if 24: end for

### 3.3. Data Preprocessing

Preprocessing is an essential initial stage before the implementation of any processing or modeling methods on the data. Data preparation is the process of converting unprocessed data into a refined and practical structure to guarantee the research’s quality and efficiency. A PPG signal’s quality is influenced by the skin tone, SpO_2_, blood flow rate, and skin temperature of the patient at measurement. Motion artifacts, power line interference, muscle artifacts, high-frequency artifacts, and low amplitude will all impact the signal strength. Hence, it is imperative to use preprocessing methods to eliminate the noise involved in a signal that could impact the extraction of features and the overall measurement of BGLs. Finally, the obtained signal is provided as the input to the module responsible for preprocessing. A representation of the input signal is given by Equation (1).

(1) $$a\left(n\right)=\left\{\left.a_{1,\;}\;a_{2,\;}a_{3,}\ldots a_{n\;}\right|0<n<S_{w}\right\}$$

Here, $a\left(n\right)$ represents the input signal, where $a_{1}$ to $a_{n}$ indicate the number of signals and $S_{w}$ indicates the window size. The preprocessing filters include the Savitzky–Golay filter and the moving average filter. Baseline wandering is eliminated by the application of the moving average filter. Noise and other artifacts are eliminated by the application of the Savitzky–Golay filter. This filter is alternatively referred to as the least squares smoothing filter or the digital smoothing polynomial filter. This technique maintains the pulse form and the high-frequency elements of the data during the process of smoothing them [24,25,26,27,28,32,33]. The filter’s output was expressed using Equation (2).

(2) $$b_{s}\left(i\right)=\frac{1}{2N+1}\left[b\left(i+N\right)+b\left(i+N-1\right)+\ldots +b\left(i-N\right)\right]$$

Here, $b_{s}\left(i\right)$ represents the input signal’s smoothed value as an output of the moving average filter, where $b\left(i\right)$ indicates the signal’s real value, N represents the parameter of window size, and 2N+1 indicates the total points in the average. The Savitzky–Golay and moving average filters improve signal quality by effectively reducing noise while preserving important features of the PPG signals, which is crucial for accurate glucose prediction. Unlike simpler filters, such as low-pass or high-pass filters, which can distort the signal’s edges or remove key features, the Savitzky–Golay filter applies polynomial smoothing that retains the integrity of the pulse amplitude and other critical trends. The moving average filter, while computationally efficient, smooths out high-frequency noise and short-term fluctuations caused by sensor artifacts or motion, improving the signal-to-noise ratio. Compared to alternatives like Gaussian or median filters, these methods offer better performance in terms of real-time signal processing and accuracy for preprocessing in non-invasive glucose estimation.

### 3.4. Feature Extraction

Feature extraction is an essential procedure that occurs after data preprocessing in DL models. It includes transforming raw data into the collection of significant and useful characteristics that can be utilized for modeling. The physiological changes are quantified based on the curve of the PPG signal. These parameters serve as the foundation for feature selection. The module’s output is a feature vector (FV) including every feature associated with the statistical fluctuations in PPG. FV comprises local features calculated at the frame level and global features calculated at the window level. The subsequent segment explains the details of feature extraction for the PPG signal.

*AR:* The coefficients of AR represent the spectrum envelope of the PPG signal. These equations quantify the alteration in a pulse’s form caused by variations in blood circulation across the blood vessels, including veins, arteries, and capillaries. AR coefficients also represent the form of the fundamental pulse. The structure of the AR power spectrum of the specific pulse must accurately correspond to the periodogram of the PPG indication signal.

(3) $$PS\left(f\right)=\frac{∆t}{N}{\left|\sum_{n=0}^{N-1}a_{n\;}e^{-j2\pi fn}\right|}^{2}$$

$PS\left(f\right)$ indicates the power spectrum of AR at different frequencies for a pulse signal. ∆t indicates the sampling interval, N is the total samples, $a_{n}$ is the AR coefficient, and $e^{-j2\pi fn}$ is the complex exponential variable. PS(f) is computed using Equation (3). For the provided periodogram, the 5th-order AR model is chosen, and AR coefficients are calculated using Yule–Walker equations. These coefficients are produced from sample covariances as specified in Equation (4) for 1 > 0.

(4) $$\sum_{k=1}^{N}c_{k}\gamma_{aa}\left[l-k\right]=-\gamma_{aa}\left[l\right]$$

N is the order of the AR model, $c_{k}$ represents the weights applied to the past values, and $\gamma_{aa}\left[l\right]$ is the autocovariance of the signal. An FV ${FAR}_{ppg}$ is formed by extracting five coefficients from Equation (4) and representing them as $F_{1},F_{2},F_{3},F_{4},$ and $F_{5}$.

(5) $${FAR}_{ppg}=\left\{{AR}_{ppg}F_{1},{AR}_{ppg}F_{2},{AR}_{ppg}F_{3},{AR}_{ppg}F_{4},{AR}_{ppg}F_{5}\right\}$$

KTE: KTE is a widely recognized technique employed to determine a signal’s energy profiles containing components of a periodic signal. Its property enables the identification of whether the signal might be classified as a noisy or clear signal. A periodic waveform is considered a clean signal if its mean value is high. Conversely, a low mean value suggests the existence of transients, noise, or artifacts in the signal. These profiles of energy are calculated both at the frame and window levels. At the frame level, the energy was calculated utilizing Equation (6). In terms of real-valued signals, the KTE operator is defined using Equation (6).

(6) $$KTE\left(t\right)={a(t)}^{2}-a(t+1)a(t-1)$$

$KTE\left(t\right)$ represents the signal’s Teager energy at time *t*; a(t) is the signal’s amplitude. Using Equation (7), the Teager energy at the window level is calculated.

(7) $$KTE\left(t\right)=S_{w}{\left(t\right)}^{2}-S_{w}(t+1)S_{w}(t-1)$$

The Teager energy for the *n*th frame was calculated at the frame level using Equation (8). $S_{fr}$ is the signal amplitude at a time in the *n*th frame.

(8) $${KTE}_{n}\left(t\right)=S_{fr}^{2}\left(t,n\right)-S_{fr}^{2}\left(t+1,n\right)S_{fr}^{2}\left(t-1,n\right)$$

Quantitative metrics like mean KTE, skewness, interquartile range, and variance were calculated for every frame. The statistical mean values of these parameters for *n* frames were calculated in the following manner (Equation (9)):

(9) $${KTE}_{fr}=\left\{{KTE}^{\alpha },{KTE}^{\beta },{KTE}^{ir},{KTE}^{sk}\right\}$$

The FV of KTE comprises the following components (Equation (10)):

(10) $$FVKTE=\left\{{KTE}_{AR},{KTE}_{n}^{\alpha },{KTE}_{n}^{\beta },{KTE}_{n}^{ir},{KTE}_{n}^{sk}\right\}$$

HRS: A functional correlation exists between deregulated glucose levels caused by heart rate variability (HRV) and diabetes. HR variability is quantified as a feature and calculated at both the window and frame levels. The computation of this characteristic involves identifying peaks within a signal window. The statistical measures are calculated for the whole statistical window. Therefore, the FVHRS is expressed using Equation (11).

(11) $$FVHRS=\left\{{HRS}_{n}^{\alpha },{HRS}_{n}^{\beta },{HRS}_{n}^{ir},{HSR}_{n}^{sk}\right\}$$

SE: The SE is the numerical value derived by calculations of an entropy function using the normalized power spectrum. This feature quantifies the attenuation of magnetic pulses, the existence of noise, and the harmonic components of the spectral form. The calculation of the power spectrum of the nth frame involves the application of a short-time FT and subsequent normalization by the squared amplitude of each bin with respect to the frame’s total power. The analysis of $A^{n}$ is expressed by Equations (12) and (13).

(12) $$A^{n}=FFT(S_{fr}\left(\tau ,n\right),L_{FFT})$$

(13) $${PF}_{a}^{n}\left[K\right]=\frac{{\left|A^{n}[K]\right|}^{2}}{\sum_{j=1}^{L_{FFT}}{\left|A^{n}[K]\right|}^{2}}\;k=1\ldots L_{FFT}\;\;$$

Ultimately, the SE is calculated by utilizing power spectral density, as shown by Equation (14).

(14) $$H_{n}^{s}=\sum_{k=1}^{L_{FFT}}{PF}_{a}^{n}\left[K\right]log({PF}_{a}^{n}\left[K\right])$$

For each of the n frames, statistical metrics are calculated, like the KTE frame evaluations. The FV FVHS denotes the mean magnitude of spectral entropy parameters (Equation (15)).

(15) $$FVHS=\left\{{HS}_{n}^{\alpha },{HS}_{n}^{\beta },{HS}_{n}^{ir},{HR}_{n}^{sk}\right\}$$

EP of the PPG signal: The log energy of the PPG signal is computed at the frame level to accurately measure respiratory rate and inform the changes (Equation (16)).

(16) $${LogEP}_{n}=\sum_{\tau =1}^{L_{Fr}}S_{fr}^{2}(\tau ,n)$$

Log En is the mathematical representation of variations in the energy of PPG in relation to the heart rate. Furthermore, the statistical elements such as interquartile range and variance were calculated. Accordingly, the FVLogEP feature vector is derived as follows in Equation (17):

(17) $$FVLogEP=\left\{Log{EP}_{n}^{AR}F_{1},Log{EP}_{n}^{AR}F_{2},Log{EP}_{n}^{AR}F_{3},Log{EP}_{n}^{AR}F_{4},Log{EP}_{n}^{AR}F_{5}Log{EP}_{n}^{\beta },Log{EP}_{n}^{ir}\;\right\}$$

PTT: PTT is a time difference between the beginning of 2 consecutive pulses. PTT was derived as follows in Equation (18):

(18) $$PTT=TZ_{2}-TZ_{1}$$

$TZ_{1}$ represents the initial point of the pulse, whereas $TZ_{2}$ represents the corresponding end point. It is estimated at the frame level, and its average statistical parameters for *n* frames were additionally determined. These parameters are expressed by the FVPTT as follows in Equation (19):

(19) $$FVPTT=\left\{{PTT}_{n}^{\alpha },{PTT}_{n}^{\beta },{PTT}_{n}^{ir},{PTT}_{n}^{sk}\right\}$$

PPI: The time interval between the two highest points of successive pulses is known as the PPI. As both indicate a whole heart cycle, the R-R interval in the signal is strongly correlated with the interval. The PPI is a method utilized to assess the cardiac status in PPG signals. A frame-level calculation is performed to determine the peak-to-peak interval. Following the computation of average statistical parameters for *n* frames, the FV is denoted as follows in Equation (20):

(20) $$FVPPI=\left\{{PPI}_{n}^{\alpha },{PPI}_{n}^{\beta },{PPI}_{n}^{ir},{PPI}_{n}^{sk}\right\}$$

PA: The systolic amplitude, also referred to as the PA of the pulse, is the pulse’s maximum value. The systolic amplitude is a measure of periodic variations in blood volume resulting from the circulation of arterial blood through the measurement location. A correlation has been shown between systolic amplitude and stroke volume [32]. Therefore, the PA is determined at the frame level, and the average statistical elements are also calculated over *n* frames. The FVPA feature vector is represented in Equation (21).

(21) $$FVPA=\left\{{PA}_{n}^{\alpha },{PA}_{n}^{\beta },{PA}_{n}^{ir},{PA}_{n}^{sk}\right\}$$

Thus, the FV consists of frequency and time domain features and is expressed by Equation (22).

(22) $$FV=\left\{{FAR}_{ppg},FVKTE,FVHRS,FVHS,FVLogEP,FVPTT,FVPPI,FVPA\right\}$$

Algorithm 2 outlines the data preprocessing and feature extraction stages necessary for analyzing photoplethysmography (PPG) signals. First, each participant’s data are processed with a moving average filter (MAF) to smooth the signal, followed by a Savitzky–Golay filter (SGF) to retain essential signal characteristics, resulting in a refined signal, d. Feature extraction then involves calculating several statistical and physiological metrics for each filtered signal, including Autoregressive (AR) coefficients, kurtosis-based features (KTE), heart rate variability (HRV), sample entropy (SE), logarithmic entropy power (EP), pulse transit time (PTT), and pulse pressure index (PPI). Each set of extracted features is saved as an individual vector, which is then combined into a comprehensive feature vector (FV). Finally, the combined feature vector dataset is split into training and testing sets with an 80:20 ratio to support model training and validation.
**Algorithm 2.** Data Preprocessing and Feature Extraction1: Data Preprocessing 2: for each d in participant data do 3:     s ← MAF(PPG) {moving average filter} 4:     d′ ← SGF(s) {Savitzky–Golay filter} 5:     X ← a(n) 6: end for 7: Feature Extraction 8: for each d′ do 9:     AR ← CalcAR(d′) 10:    Fppg ← {AR.F 1, AR.F 2, AR.F 3, AR.F 4, AR.F 5} 11:    KTE ← CalcKTE(d′) 12:    FKT E ← {KTE.µ, KTE.skew, KTE.IQR, KTE.σ} 13:    HRV ← CalcHRV(d′) 14:    FHRV ← {HRV.α, HRV.β, HRV.IQR, HRV.skew} 15:    SE ← CalcSE(d′) 16:    FSE ← {SE.α, SE.β, SE.IQR, SE.skew} 17:    EP ← CalcLogEP(d′) 18:    FLogEP ← {EP.AR.F 1, EP.AR.F 2, EP.AR.F 3, EP.AR.F 4, EP.AR.F 5, EP.β,       EP.IQR} 19:    PTT ← CalcPTT(d′) 20:    FP T T ← {PTT.α, PTT.β, PTT.IQR, PTT.skew} 21:    PPI ← CalcPPI(d′) 22:    FP P I ← {PPI.α, PPI.β, PPI.IQR, PPI.skew} 23:    FV ← Combine(Fppg, FKT E, FHRV, FSE, FLogEP, FP T T, FP P I ) 24: end for 25: Data Splitting 26: TR, TE ← Split(FV, 0.8) {80:20 Split}

### 3.5. Deep Sparse Capsule Network

In this research, the prediction process is performed by utilizing DSCNet. Current deep learning (DL)-based glucose prediction methods often face several limitations, including poor handling of noisy and artifact-prone data, difficulty in capturing complex temporal relationships in signals, and insufficient generalization ability when exposed to diverse datasets. These issues result in reduced accuracy and reliability of the glucose estimation models, especially when using physiological signals like PPG, which can be affected by noise, motion artifacts, and individual variability. DSCNet captures spatial hierarchies and relationships between features more effectively than traditional neural networks. Capsules encode both the magnitude and orientation of features, allowing for richer and more robust representations, especially in handling complex patterns like PPG signals.

**Figure 5:**
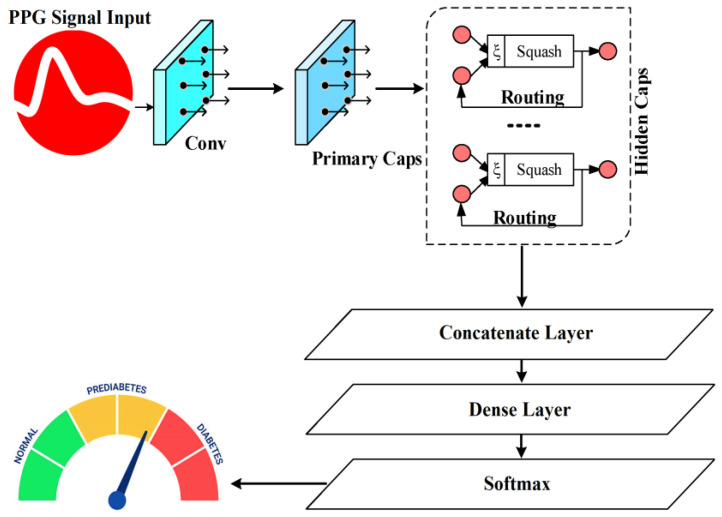
Structure of capsule networks.

In contrast to the softmax functions or conventional sigmoid that depend on a single neuron (scalar) to model the probability of classification, this work employs the length of a capsule (vector) to quantify the probability of classification and an associated category in various scenarios, depending on the capsule orientation. Employing capsule length will result in a more potent expression compared to conventional softmax or sigmoid functions. Capsule networks (CNs) differ from CNNs by using a vector-form capsule for the input and output layers, together with a dynamic routing algorithm in place of the pooling basic functions. The vector representing the capsule is still used in a few convolution levels throughout the network, with a capsule layer (CL) only being used in the final layer. Figure 5 displays the structural model of the CN. With a step size of 1, a convolution module with 256 × 9 × 9 convolution kernels makes up the first layer. The adjusted linear element function was chosen as an activation function, while the ReLU function was a continuous linear piecewise function, expressed as follows in Equation (23):

(23) $$ReLU\left(y\right)=\left\{\begin{array}{c} y,\;\;\;\;\;\;\;y>0\\ 0,\;\;\;\;\;\;\;y\leq 0 \end{array}\right.$$

The fundamental objective of the layer is to decrease the dimensionality of the input vector and extract its inherent characteristics. The input vector undergoes conditioning in an original convolution layer to generate a primary CL. The 2nd is the main CL, consisting of 32 filters measuring 9 × 9 × 256 and 8 convolution operations performed in 2 stages. Following the convolution, the data are transformed into 2-dimensional data, with each element being a vector of dimensions 1 × 8. The digital capsule makes up the third layer. The output of the CL is used as input, and the 8-dimensional capsule is converted into a 16-dimensional capsule using the transformation matrix transform methodology. The capsules include a 1-dimensional vector, and every capsule corresponds to a characteristic of a PPG signal. The class discriminant value can be evaluated by comparing the capsule outputs of different dynamic routing algorithms. Subsequently, the input vector that corresponds to the output vector’s direction is identified, while the local features are considered based on the various weights applied to the overall characteristics. This paper presents the calculation procedures for the CL in Equation (24).

(24) $$S_{r}=\sum_{q}C_{qr}{\hat{u}}_{r\left|q\right.}$$

where ${\hat{u}}_{r\left|q\right.}$ and $u_{q}$ are the high- and low-level features, $W_{qr}$ stands for a weight matrix, $S_{r}$ is the function squash’s input variable, and $C_{qr}$ is derived by dynamic routing. Iterative routing algorithms are a type of dynamic routing algorithm. DSCNet is an approach for unsupervised feature learning that aims to optimize the distribution properties of features. To obtain effective feature expressions, one can directly optimize the sparsity of the sample’s feature mapping. This optimization ensures that the features are sparse inside each sample, sparse between samples, and uniform across all samples. This is achieved by imposing joint constraints of the L1 norm and L2 norm.

Typically, the $W_{qr}$ in the capsule networks and the other convolution parameters in the entire network are updated using standard backpropagation. DSCNet substitutes the conventional backpropagation method with sparse filtering to repeatedly update $W_{qr}$, enabling the realization of an unsupervised capsule network structure. The concept of the sparse filtering update weights was defined as follows: high-level features are obtained by extracting low-level features, which is represented as follows in Equation (25):

(25) $${\hat{u}}_{r\left|q\right.}=W_{qr}u_{q}$$

Normalized matrices O and R are derived by applying column row and normalization to the matrix ${\hat{u}}_{r\left|q\right.}.$ The total absolute values of all the items in the set ${\hat{u}}_{r\left|q\right.}$ are computed. The precise procedure involves normalizing each feature to possess an equivalent activation value, dividing each feature by its L2norm over all the samples, and then normalizing the feature. A row of a matrix can be denoted as follows in Equation (26):

(26) $$R=\frac{{\hat{u}}_{r\left|q\right.}}{\left\|{\hat{u}}_{r\left|q\right.}\right\|2}=\frac{{\hat{u}}_{r\left|q\right.}}{\sqrt{\sum_{m=1}^{M}{\hat{u}}_{r\left|q\right..m}^{2}}}$$

The expression for column normalization of the characteristic matrix was defined as follows in Equation (27):

(27) $$O=\frac{R}{\left\|R\right\|2}=\frac{R}{\sqrt{\sum_{n=1}^{N}R_{nr}^{2}}}$$

where N and M are the dimensions of the eigenvectors and the number of signal types. The matrix normalization transformation ensures that every feature is equivalent to an activation value, thereby enabling it to be placed on a unit sphere surface of 2 norms [32]. Considering the analysis reported above, the objective function was expressed using Equation (28). The pseudocode of the DSCNet model is presented in Algorithm 3. Algorithm 4 describes the DSCNet model with self-attention.

(28) $$\underset{\arg W_{\mathrm{qr}}}{\min}L\left(W_{qr}\right)=\sum_{q=1}^{N}\sum_{r=1}^{M}O$$

**Algorithm 3.** DSCNet Model1: Default configurations: 2: num layers ← 4 4: kernel size ← 3 5: activation function ← ‘relu’ 6: dropoutrate ← 0.5 7: Initialize the model 8:   model ← initialize model() 9: for i = 1 to num layers do 10:  model.add layer (Conv2D(num filters[i], kernel size, activation = activation      function)) 11:  model.add layer (MaxPooling2D(pool size = 2)) 12:  model.add layer (Dropout(dropout rate)) 13: end for 14: model.add layer (Flatten()) 15: model.add layer (Dense(128, activation=activation function)) 16: model.add layer (Dropout(dropout rate)) 17: model.add layer (Dense(1, activation = ‘linear’))

**Algorithm 4.** DSCNet Model with Self-Attention1: Default configurations: 2: num layers ← 4 4: kernel size ← 3 5: activation function ← ‘relu’ 6: dropoutrate ← 0.5 7: Initialize the model 8:   model ← initialize model() 9: for i = 1 to num layers do 10:  model.add layer (Conv2D(num filters[i], kernel size, activation = activation     function)) 11:  model.add layer (MaxPooling2D(pool size = 2)) 12:  model.add layer (Dropout(dropout rate)) 13:  model.add_layer(SelfAttention()) 14: end for 15: model.add layer (Flatten()) 16: model.add layer (Dense(128, activation = activation function)) 17: model.add layer (Dropout(dropout rate)) 18: model.add layer (Dense(1, activation = ‘linear’))

## 4. Results and Discussion

### 4.1. Experimental Setup

This section outlines the results of the experiments conducted using the DSCNet research model, implemented in PyTorch (version 2.6.0) and Python (version 3.13.2). The sensor data collected were utilized to evaluate the experimental outcomes. The experiments were performed on a machine equipped with a Core i7-620M CPU, running a 64-bit version of the Windows 10 operating system, and equipped with 32 gigabytes of RAM.

### 4.2. Performance Metrics

The performance efficiency of the DSCNet model is assessed using the following metrics: Five parameters, namely MAPE, MAE, MSE, RMSE, MARD, and R^2^, were essentially employed to assess the proposed model’s ability to monitor the BGL values. Consequently, the performance metrics were evaluated using the subsequent metrics. The MAE measures the average absolute differences among the actual and predicted values computed across the dataset. The MAE measures the accuracy of the model’s performance on the same scale, as the final objective remains unchanged. A model is considered more accurate as the MAE approaches zero in Equation (29).

(29) $$MAE=\frac{1}{n}\sum_{i=1}^{n}\left(y_{i}-\hat{y}\right)$$

The MSE measures the difference between expected ($\hat{y}$) and actual values ($y_{i}$) by calculating the square root of the mean variance across the entire dataset in Equation (30).

(30) $$MSE=\frac{1}{n}\sum_{i=1}^{n}{\left(y_{i}-\hat{y}\right)}^{2}$$

The RMSE is the error value obtained by taking the square roots of the MSE in Equation (31).

(31) $$RMSE=\sqrt{\frac{1}{n}\sum_{i=1}^{n}{\left(y_{i}-\hat{y}\right)}^{2}}$$

The MAPE is the Mean Absolute Percentage Error in Equation (32).

(32) $$MAPE=\frac{100\%}{N}\sum_{i=1}^{N}\left|\frac{\hat{Y_{i}}{-Y}_{i}}{Y_{i}}\right|$$

The R^2^ is the coefficient of determination in Equation (33).

(33) $$R^{2}=1-\frac{{\sum_{i=1}^{n}\left(y_{i}-\hat{y}\right)}^{2}}{{\sum_{i=1}^{n}\left(y_{i}-\bar{y}\right)}^{2}}$$

The MARD is calculated by the average difference between a device measurement and the reference measurement. ${BGL}_{est}$ is the predicted BGL value and ${BGL}_{ref}$ is the actual BGL value in Equation (34).

(34) $$MARD=\frac{1}{n}\sum_{i=1}^{n}\frac{\left|{BGL}_{est}-\right|{BGL}_{ref}}{{BGL}_{ref}}\times 100$$

### 4.3. Sample PPG Signals

**Figure 6:**
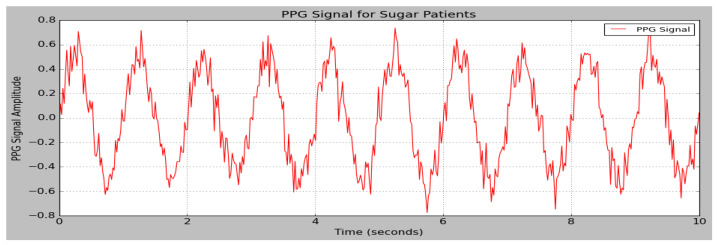
PPG signal of sugar patients.

**Figure 7:**
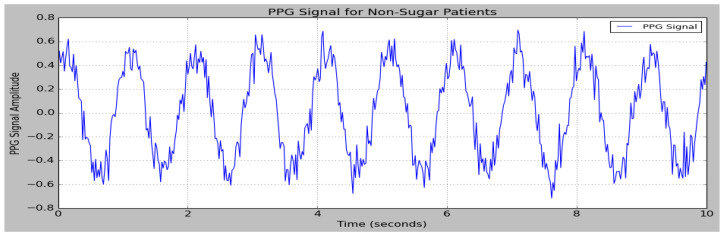
PPG signal of non-sugar patients.

The data obtained from the PPG sensor are provided as input to the research model. The sensor data are partitioned into separate testing and training sets. The performances of the DSCNet method in BGL monitoring are measured based on parameters like MAPE, MAE, MSE, RMSE, MARD, and R^2^. Figure 6. depicts the PPG signal of sugar patients, and Figure 7 depicts the PPG signals of non-sugar patients.

### 4.4. Ablation Study of DSCNet

The result comparison between the baseline DSCNet model and the enhanced DSCNet model with self-attention demonstrates significant improvements across all performance metrics.

**Figure 8:**
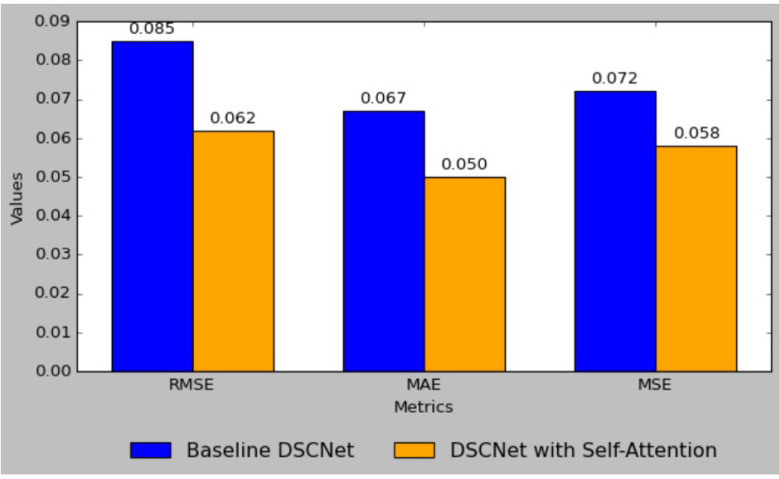
Comparison of RMSE, MAE, and MSE metrics between the baseline DSCNet and DSCNet with self-attention.

**Figure 9:**
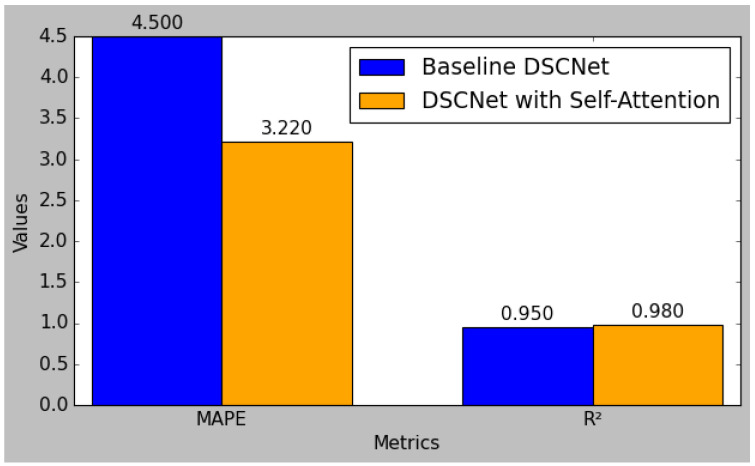
Comparison of MAPE, MAE, and R^2^ metrics between the baseline DSCNet and DSCNet with self-attention.

Figure 8 shows the comparison of the RMSE, MAE, and MSE metrics between the baseline DSCNet and DSCNet with self-attention. The baseline model achieved a Root Mean Square Error (RMSE) of 0.085, which was reduced to 0.062 in the self-attention model, reflecting a substantial 27.1% improvement. Similarly, the Mean Absolute Error (MAE) decreased from 0.067 to 0.050, marking a 25.4% enhancement, while the Mean Squared Error (MSE) was lowered from 0.072 to 0.058, indicating a 19.4% reduction. Additionally, the Mean Absolute Percentage Error (MAPE) improved from 4.50 to 3.22, representing a 28.4% decrease. The R-squared (R^2^) value also rose from 0.95 to 0.98, signifying a 3.2% increase in the model’s ability to explain variability in the data. These results collectively highlight the effectiveness of incorporating self-attention mechanisms, leading to a more robust and accurate predictive model for applications such as blood glucose level monitoring. Figure 9 shows the comparison of MAPE, MAE, and R^2^ metrics between the baseline DSCNet and DSCNet with self-attention.

**Figure 10:**
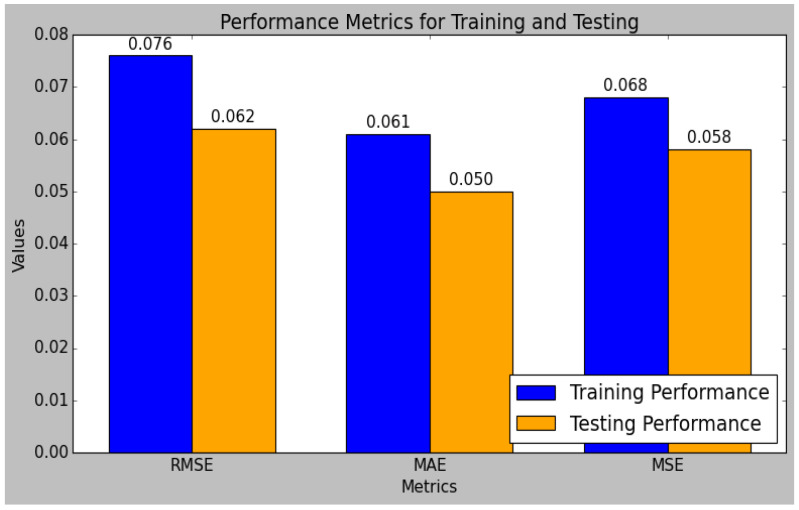
Graphical plot of research model’s training and test performance.

**Figure 11:**
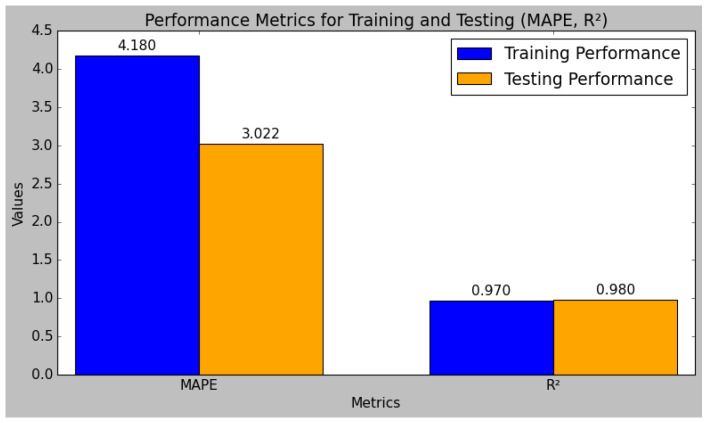
Graphical plot of research model’s training and test performance.

The research model’s performances were calculated using the training and testing sets of data that had been split from the sensor data, as shown in Table 4. In comparison to the testing set, the research model performed better in the training set. The performance evaluation of the research model reveals that it excels in both the training and testing phases, demonstrating robust predictive capabilities. The model’s R^2^ value is 0.98 during testing and 0.98 during training. Typically, the training set’s R^2^ value is higher than the test set’s, which suggests that the model is overfitting the training set. However, since the test set’s R2 is still quite high, this study’s small difference indicates that the model has not overfitted too much. Methods like regularization and cross-validation, which were included in the model training process, were used to prevent overfitting and ensure the generalizability of the model. Additionally, the MARD increases from 4.90 in training to 10.81 in testing, further validating the model’s accuracy and reliability. Overall, the model exhibits strong performance metrics, demonstrating its effectiveness and consistency. Figure 10 and Figure 11 indicate the graphical chart for the testing and training set performances of the research model.

The model achieves a low RMSE of 0.062 and an MSE of 0.058 in testing, which are lower than the training errors, indicating effective generalization to unseen data. Similarly, the MAE and MAPE improve from training to testing, with values of 0.050 and 3.022, respectively, reflecting reduced prediction errors.

### 4.5. Comparative Performance Analysis of State-of-the-Art Models

**Figure 12:**
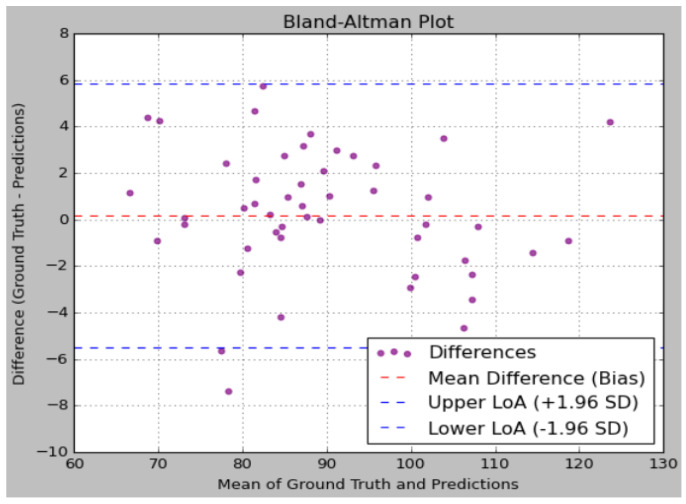
Performance analysis using Bland–Altman plot.

The DSCNet model achieves an impressive RMSE of 0.062, making it the most accurate among the compared models. This contrasts sharply with other models such as DNN, which have a considerably higher RMSE of 0.917, indicating less precise predictions. Similarly, the 1D-CNN and XGBoost models exhibit RMSE values of 12.4 and 0.298, respectively, which are significantly higher than DSCNet’s. Even the well-regarded models like CatBoost and FGSVR, with their RMSE values of 10.94 and 11.20, respectively, fall short of DSCNet’s accuracy. The low RMSE of DSCNet underscores its superior performance in minimizing prediction errors, thus establishing it as the most reliable model for glucose level estimation in this comparison, as illustrated in Figure 12 and Table 5. The DSCNet model demonstrates exceptional performance with an MAE of 0.050, which is notably superior to other models in the comparison. This low MAE indicates that DSCNet has minimal average prediction error, significantly better than models such as DNN, which have an MAE of 0.375.

Even compared to XGBoost’s MAE and the high MAE of 8.9 reported for 1D-CNN, DSCNet’s performance is far superior. Notably, the MAE achieved by DSCNet underscores its high accuracy in predicting glucose levels, outperforming existing models. Overall, DSCNet’s MAE highlights its effectiveness in delivering precise predictions with minimal error. The DSCNet model excels with an MSE of 0.058, setting a high standard in prediction accuracy when compared to other models. This MSE is substantially lower than that of DNN, which reports an MSE of 0.840, indicating a greater degree of prediction error. Even XGBoost, which has a comparatively good MSE of 0.089, does not match DSCNet’s precision. The low MSE of DSCNet underscores its superior capability to reduce prediction errors, making it the most reliable model for accurate glucose level estimation in this evaluation.

The DSCNet model demonstrates a standout performance with an MAPE of 3.022, which is significantly lower than that of other models. This MAPE value reflects DSCNet’s exceptional accuracy in predicting glucose levels as a percentage, outperforming models such as DNN with an MAPE of 5.035 and 1D-CNN, which reports an MAPE of 8.1. The substantial reduction in percentage error achieved by DSCNet highlights its effectiveness in minimizing prediction errors for glucose level estimation. The DSCNet model achieves an exceptional R^2^ value of 0.98, marking it as the top performer in terms of explaining the variance in glucose level predictions compared to other models.

This high R^2^ indicates that DSCNet can account for 98% of the variance in the data, demonstrating superior predictive accuracy. In contrast, DNN achieves an R^2^ of 0.902, while models like 1D-CNN and XGBoost have lower R^2^ values of 0.85 and 0.900, respectively. Even models like CatBoost and FGSVR, with R^2^ values of 0.92 and 0.937, respectively, do not match the predictive power of DSCNet. The high R^2^ value of DSCNet underscores its robustness and reliability in capturing and explaining the variability in glucose levels, establishing it as the most effective model in this comparison. The DSCNet model achieves an MARD of 10.81. In comparison, models such as Adaboost and 1D-CNN report MARD values of 7.3 and 8.1, respectively.

The significantly higher MARD achieved by DSCNet demonstrates its ability to deliver less effective and reliable glucose level predictions in this evaluation. The elevated value of MARD is regarded as a limitation in this study. In the future, an efficient MARD value may be attained by integrating advanced prediction algorithms for effective BGL monitoring. Consequently, this comparison indicates that the DSCNet model has achieved superior results compared to the models analyzed in this research. The key advantage of the developed research model is that it produces excellent results with minimum error values.

The use of DSCNet’s capability to accurately classify features from complicated data patterns increases the accuracy of glucose level predictions, hence improving the efficiency of the model. The findings in this research work demonstrate that the developed research model has limited data size and increased MARD value; in the future, the performance of the model can be improved by acquiring additional clinical data from medical institutions and incorporating some algorithms.

**Figure 13:**
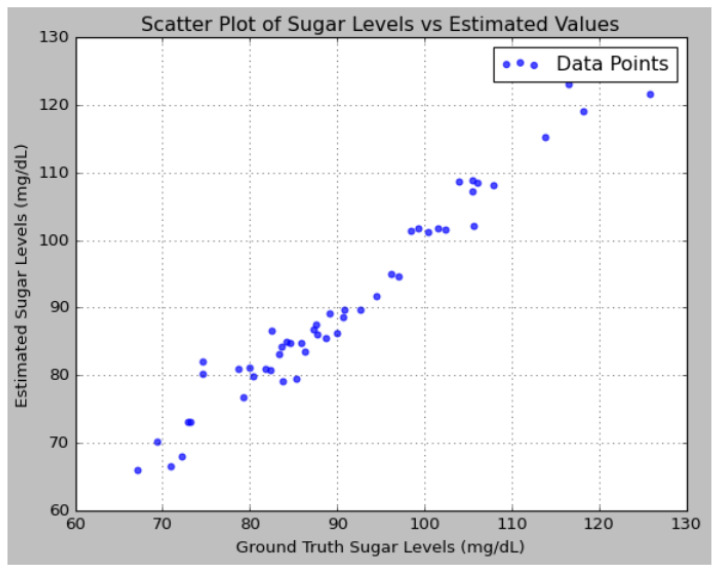
Performance analysis using scatter plot of sugar levels vs. estimated values.

An analysis of the predictions reveals that the proposed model effectively captures the underlying patterns in the sugar level data, as evidenced by the close alignment between the predicted values and the ground truth. The addition of noise to the ground truth data, simulating real-world measurement inaccuracies, allows for a more rigorous evaluation of the model’s robustness. Notably, the model’s predictions were consistently closer to the ground truth compared to the variations introduced by noise, indicating that the model possesses a high degree of accuracy. Figure 12 illustrates the performance analysis using a Bland–Altman plot. Figure 13 depicts the performance analysis of sugar levels vs. estimated values using a scatter plot.

## 5. Conclusions

In this research, a deep-learning-based DSCNet model utilizing photoplethysmography (PPG) is developed for monitoring blood glucose levels (BGLs) in individuals who prefer non-invasive methods for sugar level predictions. A small hardware module with an inbuilt PPG sensor and Raspberry Pi was designed for collecting data from the patients. To enhance the accuracy of the system, the Deep Sparse Capsule Network (DSCNet) algorithm is utilized for accurate predictions. Based on the comparison, the DSCNet model produced better outcomes with lower error values than the models that were examined in the present research. In the future, the efficiency of the model can be improved by implementing various prediction algorithms and increasing the data size.

The analysis of the predictions indicates that the proposed DSCNet model successfully captures the underlying patterns in the blood glucose level data, demonstrated by the close alignment between the predicted values and the ground truth. This reinforces the model’s effectiveness in providing accurate non-invasive sugar level predictions. Future research directions could focus on several aspects to enhance the efficacy of the DSCNet model. One potential avenue is the integration of additional physiological signals, such as electrocardiogram (ECG) or respiratory rate data, to improve the robustness and accuracy of BGL predictions. Another direction involves the exploration of advanced deep learning architectures, such as Transformer-based models or hybrid approaches that combine capsule networks with recurrent neural networks (RNNs) to capture temporal dependencies more effectively.

## Figures and Tables

**Table 1:** Comparative analysis of reviewed current models.

Ref.	Algorithm	Application Scenario	Advantages	Disadvantages
[11]	EBTA	Early diabetes detection via PPG signals	High accuracy	Complex and less interpretable
[12]	RF and XGB	Multi-wavelength PPG monitoring for health parameters	Effective for regression tasks	Slower for real-time predictions, sensitive to noise
[13]	DNN	Measuring blood components (Hb, Cr, Gl) via fingertip videos	Capable of learning complex patterns	Requires large amounts of data and computational resources
[14]	Regression model	Smartphone video-based glucose estimation	Reduces dimensionality, helps avoid overfitting	Interpretation can be challenging, sensitive to noise
[15]	RF, XGB, CB, LightGBM	Non-invasive BGL measurement via wrist PPG	High performance and speed, effective for regression tasks	Requires tuning of hyperparameters
[16]	ACNN-BiLSTM	Blood pressure measurement with multi-wavelength PPG	Utilizes multiple wavelengths of PPG signals, enhancing feature extraction and robustness	The complexity of the model may lead to overfitting, especially with smaller datasets
[17]	Adaboost	Personalized glucose monitoring system	Improves performance by focusing on difficult cases	Sensitive to noisy data and outliers
[18]	FGSVR	Wearable NIR PPG device for glucose monitoring	Effectively eliminates motion artifacts and baseline drifts, improving measurement reliability	Using only a single wavelength may restrict the sensitivity and specificity
[19]	SVM	Time–frequency analysis for BGL measurement	Effective in high-dimensional spaces, robust to overfitting	Less effective on large datasets
[20]	ML	BGL measurement across two datasets	Provides best precision values	Limited performance in complex relationships
[21]	CNN	Cohort arrangement for glucose prediction	Excellent for spatial data, can automatically learn features	Requires large labeled datasets and significant computation
[22]	RF, XGB	Estimation of glycated hemoglobin using PPG	Reduces overfitting and robust to noise	Less effective with imbalanced data
[23]	BPNN	Dual-wavelength PPG with impedance for BGL estimation	Capable of capturing complex patterns	Sensitive to overfitting
[29]	LR, SVR, RFR	Estimating hemoglobin and glucose from fingertip videos	Effective in high-dimensional spaces	Less interpretable and require more data for effective training
[30]	XGB	Simulation-based BGL estimation via PPG	Efficient with large datasets	Requires careful tuning of hyperparameters

**Table 2:** Category of blood glucose types with ranges.

Category	BG Range (mmol/L)	Level/Type
C1	3.9–6.1	Normal
C2	6.2–7.8	Prediabetic
C3	7.9–11	Diabetic

**Table 3:** Samples of collected data.

Subject ID	Gender	Age	Weight	Fasting	Non-Fasting
1	Female	27	48	5.2	5.6
5	Female	43	72	6.8	7.5
10	Male	32	65	5.7	6.4
32	Male	71	89	8.5	9.1
67	Male	55	78	10.3	12.6

**Table 4:** Research model’s training and testing performances.

Parameters	Training Performance	Testing Performance
RMSE	0.076	0.062
MAE	0.061	0.05
MSE	0.068	0.058
MAPE	4.18	3.022
R^2^	0.97	0.98

**Table 5:** Performance analysis comparison.

Model	RMSE	MAE	MSE	MAPE	R^2^	MARD
DNN [13]	0.917	0.375	0.840	5.035	0.902	-
CatBoost [15]	10.94	8.01	0.072	6.04	0.92	-
ANN-BiLSTM [16]	8.74	3.44	-	-	0.85	-
FGSVR [18]	11.20	-	-	-	0.937	7.62
Decision tree [20]	0.0856	0.0789	-	-	-	10.64
1D-CNN [21]	12.4	8.9	-	8.1	-	-
XGBoost [22]	0.298	-	0.089	-	0.900	-
BPNN [23]	6.38	5.08	0.077	-	-	4.43
MGGP [29]	0.520	0.324	0.270	-	0.881	-
Proposed model (DSCNet)	0.062	0.05	0.058	3.022	0.98	10.81

## Data Availability

Data are contained within this article.
